# Mating structures for genomic selection breeding programs in aquaculture

**DOI:** 10.1186/s12711-016-0224-y

**Published:** 2016-06-24

**Authors:** Anna K. Sonesson, Jørgen Ødegård

**Affiliations:** Nofima AS, P.O. Box 5010, 1432 Ås, Norway; AquaGen AS, P.O. Box 1240, Sluppen, 7462 Trondheim, Norway

## Abstract

**Background:**

In traditional family-based aquaculture breeding, each sire is mated to two dams in order to separate the sire’s genetic effect from other family effects. Factorial mating involves more mates per sire and/or dam and result in more but smaller full- and/or half-sib families. For traits measured on sibs of selection candidates, factorial mating increases intensity of selection between families when selection is on traditional best linear unbiased prediction (BLUP) estimated breeding values (*TRAD*-*EBV*). However, selection on genome-wide estimated breeding values (*GW*-*EBV*), uses both within- and between-family effects and the advantage of factorial mating is less obvious. Our aim was to compare by computer simulation the impact of various factorial mating strategies for truncation selection on *TRAD*-*EBV* versus *GW*-*EBV* on rates of inbreeding, accuracy of selection and genetic gain for two traits, i.e. one measured on selection candidates (*CAND*-*TRAIT*) and one on their sibs (*SIB*-*TRAIT*).

**Results:**

Sire:dam mating ratios of 1:1, 2:2 or 10:10 were tested with 100, 200 or 1000 families produced from a constant number of parents (100 sires and 100 dams), and a mating ratio of 1:2 with 200 families produced from 100 sires and 200 dams. With *GW*-*EBV*, changing the mating ratio from 1:1 to 10:10 had a limited effect on genetic gain (less than 5 %) for both *CAND*-*TRAIT* and *SIB*-*TRAIT*, whereas with *TRAD*-*EBV*, selection intensity increased for *SIB*-*TRAIT* and genetic gain increased by 41 and 77 % for schemes with 3000 and 12,000 selection candidates, respectively. For both *GW*-*EBV* and *TRAD*-*EBV*, rates of inbreeding decreased by up to ~30 % when the mating ratio was changed from 1:1 to 10:10 for schemes with 3000 to 12,000 selection candidates. Similar results were found for alternative heritabilities of *SIB*-*TRAIT* and total number of tested sibs.

**Conclusions:**

Changing the sire:dam mating ratio from 1:1 to 10:10 increased genetic gain substantially with *TRAD*-*EBV*, mainly through increased selection intensity for the *SIB*-*TRAIT*, whereas with *GW*-*EBV*, it had a limited effect on genetic gain for both traits. Rates of inbreeding decreased for both selection methods.

## Background

In selective breeding, the procedures used to select parents and mate the selected parents are important for achieving genetic progress. In traditional family-based aquaculture breeding programs, the number of tanks (full-sib families) is a cost limitation. Many of the traditional family-based aquaculture breeding programs apply a sire:dam mating ratio of 1:2, i.e. each sire is mated to two dams in order to separate the sire’s genetic effect from other family effects, tank effects (since families are kept in separate tanks until physical tagging), maternal effects (e.g. egg quality), and dominance genetic effects. For aquaculture species, control of reproduction is possible by artificial stripping of eggs and milt, and natural mating can occur and result in mating ratios that differ from 1:1, i.e. factorial mating. Woolliams [1] showed that factorial mating designs increase genetic gain without increasing inbreeding compared to a 1:1 sire:dam mating ratio for schemes using truncation selection on traditional best linear unbiased prediction (BLUP) breeding values with a constant number of parents.

For aquaculture populations, traits that require invasive phenotyping methods (carcass and disease resistance traits) are necessarily measured on sibs of the candidates. Usually, there are 15 to 100 test individuals used per family to estimate the breeding values of the untested candidates. However, with factorial mating designs in which the number of mates per sire and/or dam is larger, a potential reduction in the number of full-sibs is compensated by larger numbers of paternal and maternal half-sibs.

The standard criteria used in selective breeding are BLUP estimated breeding values (EBV) based on phenotype and pedigree data [2]. For traits that cannot be measured on selection candidates, but that are instead measured on sibs of candidates, traditional BLUP assigns identical breeding value predictions to all non-phenotyped members of a family. However, if genome-wide estimated breeding values [3] are used, different breeding values are obtained for each individual within a family and thus both within- and between-family genetic variation are used for selection. As a consequence, less emphasis is given to the between-family component [4]. Therefore, with traditional BLUP estimated breeding values, factorial mating is expected to increase the intensity of selection between families on traits that are measured on sibs of the candidates (since more families are available for the same number of parents). However, for genome-wide estimated breeding values, even for traits that are measured on sibs of the candidates, selection is done both within and between families and the advantage of factorial mating is less obvious.

Our aim was to compare by computer simulation the impact of various factorial mating strategies for truncation selection on traditional BLUP EBV versus genome-wide EBV on rates of inbreeding, accuracy of selection and genetic gain for two traits, i.e. one measured on selection candidates and one on their sibs. We applied random mating of sires and dams among the selected sires and dams within defined mating ratios.

## Methods

### Simulation of the historical population

A historical population with an effective population size (*N*_*e*_) of 1000 was simulated for 4000 generations according to Fisher–Wright’s population model [5, 6]. Five hundred males and 500 females were randomly selected and mated using sampling with replacement. The last generation of these 4000 generations was used as the first generation (*Generation0*) of the selection population of the breeding scheme.

### Simulation of the breeding scheme between *Generation0* and *Generation10*

Between *Generation0* and *Generation10*, the selection population was simulated as follows. Individuals were split into one group of selection candidates of 3000, 6000 or 12,000 (*Ncand*) and one group of 3000 or 1000 test individuals (*Ntest*). Two traits were considered: *CAND*-*TRAIT*, a trait measured on the selection candidates and *SIB*-*TRAIT*, a trait that requires an invasive method and was measured on test individuals, which were assumed to be sacrificed during the recording process. Parents were selected from among the selection candidates based on their respective traditional BLUP (*TRAD*-*EBV*) or genome-wide (*GW*-*EBV*) estimated breeding values. For the *GW*-*EBV*-based schemes, the *Ncand* group constituted the reference population for *CAND*-*TRAIT* and the *Ntest* group constituted the reference population for the *SIB*-*TRAIT* (see Table 1 for the respective tested family sizes).

**Table 1:** Family sizes used in the simulated schemes (numbers of candidates or sibs)

Sire:dam mating ratio	3000	6000	12,000
1:1, i.e. 100 families	30	60	120
2:2, i.e. 200 families	15	30	60
1:2, i.e. 200 families	15	30	60
10:10, i.e. 1000 families	3	6	12

One hundred sires and 100 dams were selected with varying sire:dam mating ratios of 1:1, 2:2 or 10:10 that produced 100, 200 or 1000 full-sib families, respectively. With a mating ratio of 1:2, 100 sires and 200 dams were selected that produced 200 families. The *Ncand* and *Ntest* were split equally over the full-sib families (see Table 1 for family size). Throughout this paper, we define factorial mating as designs that apply sire:dam mating ratios that differ from 1:1, i.e. where one sire is mated to several dams and/or one dam is mated to several sires.

### Genome

Briefly, the genome structure of all individuals was diploid with ten chromosomes, each with a size of 100 cM (see [7] for more details). The infinite sites mutation model [8] was used to create new bi-allelic single nucleotide polymorphisms (SNPs), using a mutation rate of 10^−8^ per nucleotide [9] and assuming 1,000,000 nucleotides per cM. SNPs followed Mendel’s law of inheritance, and the Haldane mapping function [10] was used to simulate recombination events. For each trait, 50 SNPs per chromosome were sampled randomly to be quantitative trait loci (QTL) (sampling without replacement from SNPs with a minor allele frequency (MAF) >0.05, resulting in an average MAF of 0.17). From the remaining SNPs, the 1000 SNPs with the highest MAF were chosen as genetic markers. The latter resulted in a total of 10,000 SNPs spread over 1000 cM. Reduced numbers of SNPs were obtained by selecting every 2nd SNP, resulting in 5000 SNPs.

Effects of the QTL alleles were sampled from a gamma distribution with a shape parameter of 0.4 and a scale parameter of 1.66 [11]. There were no pleiotropic QTL effects, and no genetic or environmental correlation between the two traits. QTL effects were assumed to be either positive or negative with a probability of 0.5, because the gamma distribution only gives positive values. After sampling, these QTL allelic effects were standardized such that the total genetic variance was equal to 1 for each trait, by calculating the standard deviation of the simulated genetic values of individuals from the last generation of the historical population, and scaling all QTL allelic effects by this standard deviation such that the genetic variance became 1.

### Calculation of phenotypic values and true and estimated traditional BLUP and genome-wide breeding values

The true genome-wide breeding values of an individual for *CAND*-*TRAIT* and *SIB*-*TRAIT* were calculated as:

$$TBV_{i(trait)} = \mathop \sum \limits_{j = 1}^{500} x_{ij} g_{j(trait)} ,$$

where $x_{ij}$ is the number of copies of allele 1 that individual $i$ has at the $j{th}$ QTL position and $g_{j(trait)}$ is the allele substitution effect of allele 1 at the $j{th}$ QTL position for each trait. The phenotypic value of an individual for trait $t$ was simulated by adding an error term sampled from a normal distribution to the true breeding value ($TBV_{i(trait)}$):

$$y_{i(trait)} = TBV_{i(trait)} + \varepsilon_{i(trait)} ,$$

where $\varepsilon_{i(trait)}$ is an error term for individual $i$, which was normally distributed $N\left( {0,\sigma_{{e\left( {trait} \right)}}^{2} } \right)$, and $\sigma_{e}^{2}$ was adjusted so that the heritability was 0.25 for *CAND*-*TRAIT* and 0.25 or 0.10 for *SIB*-*TRAIT*, which is common for such traits.

For *TRAD*-*EBV*, BLUP breeding values were calculated as in [2] by combining own performance and information of all available relatives from the current and earlier generations. The following statistical model used was:

$$y_{i(trait)} = \mu_{trait} + u_{i(trait)} + e_{i(trait)} ,$$

where $y_{i(trait)}$ is the record of individual $i$; $\mu_{trait}$ is the overall mean, i.e. the only fixed effect in the model, $u_{i(trait)}$, is the additive genetic effect of individual $i$, which was correlated across individuals following the pedigree-based relationship matrix ${\mathbf{A}}$, and $e_{i(trait)}$ is the error effect of individual $i$.

For *GW*-*EBV*, marker effects were predicted using the BLUP method as described in [3]. The statistical model used to estimate the marker effects for *CAND*-*TRAIT* and *SIB*-*TRAIT* followed method 2 of [12] and was:

$$y_{i(trait)} = \mu_{trait} + \mathop \sum \limits_{j}^{n} X_{ij} a_{j(trait)} + e_{i(trait)} ,$$

where $y_{i(trait)}$ is the record of individual $i$ for *CAND*-*TRAIT* and *SIB*-*TRAIT*; $X_{ij} = \frac{{\left( {Z_{ij} - 2p_{j} } \right)}}{{\sqrt {2p_{j} \left( {1 - p_{j} } \right)} }}$ denotes a standardized marker genotype (with mean 0 and variance 1), where $Z_{ij}$ is the original marker genotype (0, 1 or 2 alleles) and $p_{j}$ is the allele frequency at locus $j$; $a_{j(trait)}$ is the random effect of the $j{th}$ marker, with $var\left( {a_{j(trait)} } \right)$ assumed to be $1/n$, where $n$ is the number of markers (since the total genetic variance was standardised to 1); and $e_{i(trait)}$ is a random residual.

Genome-wide breeding values were estimated by summing the estimates of the marker effects $\hat{a}_{j(trait)}$:

$$EBV_{i(trait)} = \mathop \sum \limits_{j}^{n} X_{ij} \hat{a}_{j(trait)} .$$

In this model, all available information is used to estimate SNP effects, which implies that, as above, own performance and information on all available relatives from the current and earlier generations were used.

The relative economic weights for *CAND*-*TRAIT* and *SIB*-*TRAIT* in the total merit index used for selection were equal to 50 %, i.e.:

$$EBV_{i(index)} = 0.5EBV_{i(CAND - TRAIT)} + 0.5EBV_{i(SIB - TRAIT)} .$$

### Statistics

Summary statistics for each of the schemes were based on 100 replicated simulations. Selection schemes were run for ten generations (*Generation0*-*Generation10*). The breeding schemes were compared based on rates of inbreeding ($\Delta F$) in *Generation10*, genetic gain for *CAND*-*TRAIT* and *SIB*-*TRAIT* ($G$, measured as genetic change in genetic standard deviation units ($\upsigma_{a}$) from generation *Generation0* to generation *Generation10*), the percentage of total genetic gain that came from the *SIB*-*TRAIT* (% *SIB*-*TRAIT*), and accuracy of the total merit index of the selection candidates (*Acc*). Inbreeding coefficients ($F$) were calculated based on pedigree information, assuming that individuals in *Generation0* were unrelated base parents. Rates of inbreeding were calculated per generation. Accuracy of the total merit index of the selection candidates (*Acc*) was calculated as the correlation between the true and estimated breeding values.

## Results

Results from schemes with different numbers of selection candidates are in Table 2. For selection based on traditional BLUP estimated breeding values (*TRAD*-*EBV*), factorial mating (e.g. changing the mating ratio from 1:1 to 10:10 with a constant number of parents) substantially increased genetic gain for the *SIB*-*TRAIT* (from 0.17 to 0.24$\upsigma_{g}$, 0.16 to 0.23$\upsigma_{g}$, and 0.13 to 0.23$\upsigma_{g}$ for *Ncand* = 3000, 6000 and 12,000, respectively), while genetic gain for *CAND*-*TRAIT* was unchanged or only slightly reduced. Consequently, total genetic gain was greater for factorial mating designs, as was the  % *SIB*-*TRAIT*. Genetic gains with mating ratios 1:2 and 2:2 were, as expected, intermediate between those obtained with the two most extreme designs; with mating ratio 2:2, the genetic gain for *SIB*-*TRAIT* was slightly larger than with mating ratio 1:2. Despite faster genetic gain, the 10:10 mating ratio had lower rates of inbreeding than the 1:1 mating ratio (from 0.014 to 0.011, 0.015 to 0.013, and 0.008 to 0.005 for *Ncand* = 3000, 6000 and 12,000, respectively). *Acc* tended to increase when the mating ratio changed from 1:1 to 10:10.

**Table 2:** Effect of sire:dam mating ratio on genetic gains, rates of inbreeding and accuracy of selection with varying numbers of candidates

Sire:dam mating ratio	*G*_*CAND*-*TRAIT*_^a^ (σ_g_)	*G*_*SIB*-*TRAIT*_^a^ (σ_g_)	% *SIB*-*TRAIT*	∆*F* ^b^	*Acc* ^c^
*Ncand* = 3000					
*TRAD*-*EBV*					
1:1	0.36	0.17	32	0.014	0.492
2:2	0.35	0.19	35	0.014	0.503
1:2	0.34	0.16	32	0.011	0.489
10:10	0.34	0.24	41	0.011	0.510
*GW*-*EBV*					
1:1	0.37	0.35	49	0.010	0.728
2:2	0.36	0.36	50	0.010	0.718
1:2	0.35	0.33	48	0.011	0.716
10:10	0.38	0.35	48	0.011	0.711
*Ncand* = 6000					
*TRAD*-*EBV*					
1:1	0.39	0.16	29	0.015	0.501
2:2	0.38	0.20	34	0.015	0.504
1:2	0.38	0.16	30	0.011	0.506
10:10	0.39	0.23	37	0.013	0.514
*GW*-*EBV*					
1:1	0.42	0.37	47	0.008	0.754
2:2	0.42	0.37	47	0.007	0.743
1:2	0.41	0.35	46	0.005	0.738
10:10	0.43	0.37	46	0.005	0.734
*Ncand* = 12,000					
*TRAD*-*EBV*					
1:1	0.43	0.13	24	0.015	0.490
2:2	0.42	0.19	31	0.014	0.514
1:2	0.42	0.15	26	0.011	0.501
10:10	0.43	0.23	35	0.014	0.518
*GW*-*EBV*					
1:1	0.44	0.39	47	0.009	0.767
2:2	0.45	0.40	47	0.008	0.761
1:2	0.44	0.40	47	0.007	0.754
10:10	0.46	0.40	46	0.006	0.753

Genetic gain (G) for *CAND*-*TRAIT* and *SIB*-*TRAIT*, percentage of total genetic gain that comes from the *SIB*-*TRAIT* (% *SIBTRAIT*), rates of inbreeding (∆*F*) and accuracy of the total merit index of the selection candidates (*Acc*) in *Generation10* for schemes with different number of candidates; 100 sires and 100 dams were selected for schemes with mating ratio 1:1, 2:2 and 10:10 and 100 sires and 200 dams for schemes with mating ratio 1:2; heritability of *SIB*-*TRAIT* = 0.25; *Ntest* = 3000

^a^Standard errors between 0.0002 and 0.0006

^b^Standard errors between 0.000000 and 0.000006

^c^Standard errors between 0.0001 and 0.0004

Selection on genome-wide breeding values (*GW*-*EBV*) achieved higher genetic gains than selection on traditional breeding values (*TRAD*-*EBV*), especially for *SIB*-*TRAIT*. However, the most striking result of the schemes based on *GW*-*EBV* was that factorial mating generally had a limited effect on genetic gain for both traits (+0 to 3 % for *CAND*-*TRAIT*; +1 to 5 % for *SIB*-*TRAIT*), and thus also on the  % *SIB*-*TRAIT*. Rates of inbreeding were considerably lower for all genome-wide selection schemes than for traditional selection. Furthermore, when changing the mating ratio from 1:1 to 10:10, rates of inbreeding decreased from 0.015 to 0.014 and from 0.009 to 0.006 for *Ncand* = 6000 and 12,000, respectively, but not with *Ncand* = 3000. *Acc* tended to decrease when the mating ratio increased from 1:1 to 10:10.

The results for the scheme with a lower heritability (0.10) for *SIB*-*TRAIT* (*Ncand* = 6000) are in Table 3. As expected, the lower heritability resulted in a shift of genetic gain towards *CAND*-*TRAIT* (giving somewhat increased genetic gain for this trait), while genetic gain for *SIB*-*TRAIT* was substantially reduced. With *TRAD*-*EBV*, factorial mating resulted in considerably greater genetic gain for *SIB*-*TRAIT*, with increases that were of similar magnitude as for the scheme with a heritability of 0.25 for *SIB*-*TRAIT*, but had little effect on genetic gain for *CAND*-*TRAIT.* Again, rates of inbreeding were lower for the factorial mating designs (0.005 for 10:10 and 0.008 for 1:1). Also with *GW*-*EBV*, factorial mating had little effect on genetic gain, but a substantial effect on rates of inbreeding.

**Table 3:** Effect of sire:dam mating ratio on genetic gains, rates of inbreeding and accuracy of selection with a lower heritability for *SIB*-*TRAIT*

Sire:dam mating ratio	*G*_*CAND*-*TRAIT*_^a^ (σ_g_)	*G*_*SIB*-*TRAIT*_^a^ (σ_g_)	% *SIB*-*TRAIT*	∆*F* ^b^	*Acc* ^c^
*TRAD*-*EBV*					
1:1	0.44	0.05	10	0.015	0.525
2:2	0.45	0.07	13	0.014	0.526
1:2	0.43	0.04	9	0.010	0.518
10:10	0.45	0.07	13	0.011	0.514
*GW*-*EBV*					
1:1	0.51	0.14	22	0.008	0.744
2:2	0.51	0.15	23	0.007	0.731
1:2	0.50	0.15	23	0.005	0.728
10:10	0.51	0.15	23	0.005	0.715

Genetic gain (*G*) for *CAND*-*TRAIT* and *SIB*-*TRAIT*, percentage of total genetic gain that comes from the *SIB*-*TRAIT* (% *SIB*-*TRAIT*), rates of inbreeding (∆*F*) and accuracy of the total merit index of the selection candidates (*Acc*) in *Generation10* for schemes with lower heritability of *SIB*-*TRAIT* of 0.10; 100 sires and 100 dams were selected for schemes with mating ratios 1:1, 2:2 and 10:10 and 100 sires and 200 dams for schemes with mating ratio 1:2; *Ntest* = 3000; *Ncand* = 6000

^a^Standard errors between 0.0002 and 0.0006

^b^Standard errors between 0.000000 and 0.000003

^c^Standard errors between 0.0001 and 0.0002

Results for the scenario with fewer individuals in the sib test (*Ntest*) having phenotypic records on *SIB*-*TRAIT* are in Table 4. Reducing the number of sibs with phenotypic records had a similar effect as reducing the heritability of *SIB*-*TRAIT* (although the effects were less strong), since in both scenarios the amount of information on sibs is reduced, either because the information content of each sib phenotype for the *SIB*-*TRAIT* is reduced (lower heritability) or because the number of phenotypes recorded on sibs is reduced.

**Table 4:** Effect of sire:dam mating ratio on genetic gains, rates of inbreeding and accuracy of selection with fewer tested sibs

Sire:dam mating ratio	*G*_*CAND*-*TRAIT*_^a^ (σ_g_)	*G*_*SIB*-*TRAIT*_^a^ (σ_g_)	% *SIBTRAIT*	∆*F* ^b^	*Acc* ^c^
*TRAD*-*EBV*					
1:1	0.41	0.11	21	0.015	0.461
2:2	0.40	0.14	26	0.015	0.471
1:2	0.40	0.11	22	0.011	0.463
10:10	0.41	0.17	29	0.11	0.472
*GW*-*EBV*					
1:1	0.44	0.31	41	0.008	0.694
2:2	0.44	0.32	42	0.007	0.689
1:2	0.44	0.30	41	0.006	0.682
10:10	0.44	0.32	42	0.005	0.672

Genetic gain (*G*) for *CAND*-*TRAIT* and *SIB*-*TRAIT*, percentage of total genetic gain that comes from the *SIB*-*TRAIT* (% *SIB*-*TRAIT*), rates of inbreeding (∆*F*), and accuracy of the total merit index of the selection candidates (*Acc*) in *Generation10* for schemes with a sib test using fewer individuals, *Ntest* = 1000; 100 sires and 100 dams were selected for schemes with mating ratios 1:1, 2:2 and 10:10 and 100 sires and 200 dams for schemes with mating ratio 1:2; Heritability for *SIB*-*TRAIT* = 0.25; *Ncand* = 6000

^a^Standard errors between 0.0002 and 0.0006

^b^Standard errors between 0.000000 and 0.000006

^c^Standard errors between 0.0001 and 0.0004

## Discussion

The main result of this study is that with genome-wide estimated breeding values, the effect of sire:dam mating ratio on genetic gain was less than 5 % for both traits when selecting simultaneously for *CAND*-*TRAIT* and *SIB*-*TRAIT*. The offspring (total number ranging from 6000 to 15,000) were split over the 100, 200 or 1000 families, i.e. each individual had many sibs, either full-sibs or half-sibs, with the different mating ratios. In the schemes where both *CAND*-*TRAIT* and *SIB*-*TRAIT* had a heritability of 0.25 and both *Ntest* and *Ncand* were equal to 3000 (Table 2), genetic gains were similar for *SIB*-*TRAIT* and *CAND*-*TRAIT*, although only *CAND*-*TRAIT* was measured on the selection candidates. Having genomic information on both candidates and their sibs enabled within-family selection for both traits, which explains the higher genetic gain obtained for schemes based on genome-wide breeding values.

Several other studies on aquaculture species have shown that factorial mating designs were beneficial to maintain low rates of inbreeding and increase genetic gains (e.g. [13, 14]) using phenotypic selection or selection on traditional BLUP estimated breeding values and genome-wide estimated breeding values [15]. We confirmed those results, and showed that rates of inbreeding decreased by ~20 to 30 % for both traditional and genomic selection schemes when changing the sire:dam mating ratio from 1:1 to 10:10. For factorial mating systems with more mates per sire and/or dam, more (but smaller) families are produced from the same number of parents. Indeed, for the schemes in Table 2 with *Ncand* = 6000, the number of full-sib families from which parents were selected increased from 45 to 141 for schemes based on genome-wide estimated breeding values and from 24 to 84 for schemes based on traditional BLUP estimated breeding values, when the mating ratio changed from 1:1 to 10:10. Thus, in schemes with a 10:10 mating ratio, the best parents are mated with many partners, thus increasing the probability of combining favorable sires and dams, whereas in schemes with a 1:1 and to some extent a 2:2 mating ratio, the best parents can by chance mate with inferior partners. Hence, the number of superior families decreases as the number of matings per parent decreases. This explains that, in schemes based on traditional BLUP estimated breeding values, genetic gain for *SIB*-*TRAIT* was smaller for a mating ratio of 1:1 than for a mating ratio of 10:10 (since selection was only based on family means). It also explains the higher rates of inbreeding for the mating ratio 1:1 compared to 10:10, since parents are to a larger extent selected within the fewer superior families (using either random or genomic within-family selection).

Mating ratio had a small effect on the accuracy of estimated breeding values of schemes with either traditional or genome-wide breeding values when changing the mating ratio by keeping the number of parents constant but mating them to different numbers of mates. For the scheme in Table 2 with *Ncand* = 6000, *Acc* of the *TRAD*-*EBV* was equal to 0.501 and 0.514 for mating ratios 1:1 and 10:10, respectively. Hence, the increase in genetic gain with more mates per sire and/or dam in the factorial mating design was mainly due to greater selection intensity rather than accuracy, i.e. when changing the mating ratio from 1:1 to 10:10 results in 1000 instead of 100 families to select from. Thus, our findings confirmed the results of [1, 16], which showed that increasing the number of mates mainly affects selection intensity. *Acc* of the *GW*-*EBV* was equal to 0.754 and 0.734 for mating ratios 1:1 and 10:10, respectively. This decrease in *Acc* can be explained by a decrease in genetic variance during selection in earlier generations, which was not observed in *Generation2* (result not shown). Thus, with the *GW*-*EBV* schemes, increasing the number of mates per sire and/or dam in the factorial mating design led to little change in accuracy, selection intensity and genetic gain because family information is relatively less important due to the greater within-family component compared to selection on *TRAD*-*EBV*.

Overall, selection on *GW*-*EBV* increased genetic gain for *CAND*-*TRAIT* by 4 to 15 % and for *SIB_TRAIT* by 50 to 240 %, compared with selection on *TRAD*-*EBV*. When the heritabilities of *CAND*-*TRAIT* and *SIB*-*TRAIT* were identical, selection on *GW*-*EBV* led to similar genetic gains for both traits, although *SIB*-*TRAIT* was measured on sibs of the candidates only. However, with a smaller number of test individuals or a larger genome (i.e. with more chromosomes, genomic relationships between sibs become closer to their expectation of 0.5, and differences in relationships are smaller, resulting in less accurate genomic selection), genetic gain for the *SIB*-*TRAIT* would likely have been somewhat smaller.

In this study, comparisons between breeding schemes was done without a restriction on rate of inbreeding and showed that rates of inbreeding were ~50 % lower with selection on *GW*-*EBV* than on *TRAD*-*EBV*. Thus, if restrictions are imposed on rate of inbreeding rather than on the number of parents and offspring per parent, genetic gains are expected to be higher than observed here and to be higher for the schemes based on *GW*-*EBV* than on *TRAD*-*EBV*. However, the rates of inbreeding presented here were based on pedigree-based relationships, which were shown by [16] to underestimate genome-based inbreeding for schemes with selection on *GW*-*EBV*. The lower rates of inbreeding found by increasing the number of mates per sire and/or dam in the factorial mating designs should allow, in practice, for more intense selection on both traits for both traditional and genomic selection, and thus higher genetic gain.

As a test, *GW*-*EBV* schemes were also run with a fixed tank effect in the model (results not shown). Overall, similar results were obtained for all mating ratios, but all genetic gains were slightly reduced, because the number of degrees of freedom of the model was increased since more effects were fitted, and thus the accuracy of the breeding values decreased.

In this study, *CAND*-*TRAIT* and *SIB*-*TRAIT* were assumed to be uncorrelated. If the genetic correlation differs from 0, the *SIB*-*TRAIT* can be separated into a component that can be predicted by *CAND*-*TRAIT* records (which was recorded on the candidates) and a component that is uncorrelated to *CAND*-*TRAIT* and thus cannot be predicted from *CAND*-*TRAIT* records. The latter component would thus be similar to the uncorrelated *SIB*-*TRAIT* considered in this study, and the combination of *CAND*-*TRAIT* and the predictable component of *SIB*-*TRAIT* would be like the *CAND*-*TRAIT* in our study. This decomposition of the SIB-TRAIT may alter the relative importance of the two traits, but the effect of an altered importance of the *SIB*-*TRAIT* was found to be small in the current study (Table 3). Hence, our general results are not expected to be sensitive to a non-zero correlation between the CAND-TRAIT and SIB-TRAIT.

For all mating ratios, we used the same number of parents and offspring (selection candidates and test-sibs), except for mating ratio 1:2. Thus, the same number of individuals was phenotyped and genotyped and, any differences in cost are due to differences in the number of families used. For practical family-based breeding schemes, the number of families is the largest limitation, because of the high investment and running costs for each family tank, which are required to raise families until tagging size. At least 50 single-pair mated families are needed to maintain inbreeding rate within the generally recommended value of 1 % [17]. For genomic selection, separate family tanks may not be needed, because the relationships among individuals can be established based on data from genetic markers. Thus, a larger number of families and a larger total number of animals can be realized more easily, such that increased selection intensity is possible at the same rate of inbreeding. Without family tanks, it will be necessary to estimate marker effects in a separate population of sibs that are raised in addition to the candidates. Management of these two populations must be optimized such that the family contributions are similar.

## Conclusions

Changing the sire:dam mating ratio from 1:1 to 10:10 increased genetic gain substantially with *TRAD*-*EBV*, mainly through increased selection intensity for the *SIB*-*TRAIT*, whereas with *GW*-*EBV*, it had a limited effect on genetic gain for both traits. Rates of inbreeding decreased for both selection methods.
